# Net zero in power and industry—creating or destroying value and jobs?

**DOI:** 10.1016/j.isci.2025.112185

**Published:** 2025-03-10

**Authors:** Caroline Ganzer, Piera Patrizio, Niall Mac Dowell

**Affiliations:** 1 Max Planck Institute for Dynamics of Complex Technical Systems, 39106 Magdeburg, Germany; 2 Sargent Centre for Process Systems Engineering, Imperial College London, London SW7 2AZ, United Kingdom; 3 Centre for Environmental Policy, Imperial College London, London SW7 2AZ, United Kingdom

**Keywords:** Energy resources, Energy policy

## Abstract

Value and job creation are frequently claimed as positive by-products of decarbonization. The lack of a standardized methodology and quantitative analyses limit the credibility of such claims. In this work we apply the Jobs and Economic Development Impacts (JEDI) framework to our model of UK power and industry and consistently quantify the impact on economic value and employment for a range of net zero scenarios. Our results suggest that a net zero target can create gross value added and jobs under the right conditions. Offshoring industrial emissions, however, results in a reduction of both, whereas expanding domestic low-carbon industry can lead to further value and job creation. We determine economic sectors with gains and losses, and quantify the impact of local supply chains vs. importing goods and services. A thought experiment indicates ways to further increase value added.

## Highlights

• We connect a bottom-up model of power and industry with socio-economic data
• Under constant economic structure a net zero target increases value and employment
• Offshoring emissions diminishes, expanding low-carbon production elevates impacts

## Introduction

Transition pathways to net zero emissions have broad socio-economic impacts. The power and industry sectors generate gross value added (GVA) in the economy, contributing to economic growth, and provide employment opportunities. Climate change mitigation is linked with the sustainable development goals (SDGs),^1^ most closely with goal 7—affordable and clean energy, goal 8—economic growth, employment and decent work, goal 9—resilient infrastructure, sustainable industrialization, and goal 13—combat climate change and its impacts. Both trade-offs and synergies between the goals are conceivable. Conflicts can exist between labor and environmental movements, manifesting in trade-offs between saving jobs and saving the climate.^2^ This becomes apparent, for instance, in discussions around the coal sector and supply chain.^3^ Conversely, when strategies for power and industry not only mitigate climate change but also result in economic growth and job creation, synergies between the individual goals are achieved. This connection is referenced by the IPCC,^4^ and a potential for “green collar jobs” and “value of exports from the low carbon economy” has been proclaimed by the UK government.^5^ In addition, the concept of a “fair” or “just” transition is broadly recognized as being important. It is mentioned in the preamble of the Paris Agreement,^6^ the IPCC special report,^4^ and pledges from both the public and private sectors at COP26.^7^ The EU maintains a Just Transition Mechanism as well as a Just Transition Fund,^8^^,^^9^ aiming to support regions which are projected to be adversely affected by the energy transition via financing research, reskilling workers, transforming existing, and creating new firms. Just transition initiatives exist in countries around the world, many of them focusing on jobs.^10^ Substantial, sustained support from the public sector is imperative in realizing the transition. Consequently, it follows that governments may impose employment targets in addition to emissions budgets, and define value creation as additional dimension to least cost. Socio-economic benefits could be taken into account when allocating public funds. In the UK, the private sector claims to generate jobs in decarbonization efforts, however, there is no synchronized methodology across projects and sectors.^11^

Value and job creation of low-carbon technologies and energy transition scenarios have been assessed using several methods, including employment ratios/factors/values, supply chain analysis, input-output modeling, ex-post analysis of historical data, and computable general equilibrium (CGE) models.^12^^,^^13^ Some analyses distinguish between direct, indirect, and induced jobs,^14^ however, there exists variation with regard to their definitions.^15^ Generally, direct effects are associated with the construction and operation of assets, indirect effects originate within the supply chains of these assets, and induced effects result from the increased spending in the economy due to the direct and indirect effects.^16^ In the literature, the power sector has received more attention compared to the industrial sector, with a pronounced focus on the potential job creation by renewable energy sources.^13^ Furthermore, qualitative analyses and arguments appear to outnumber modeling results and quantitative analyses. One author asserts that the field lacks empirical studies in favor of analytical frameworks,^2^ another declaring the field exhibits a high degree of reusing and recycling existing data but little original research.^13^

The minority of reviewed sources present transition impacts on gross value added (GVA) or gross domestic product (GDP). Turner et al. estimate value added, GDP and job creation for industrial supply chains undergoing decarbonization, with a focus on the German cement sector. They consider offshoring industrial emissions and carbon leakage, noting it will likely displace jobs and GDP along the supply chain.^17^ More recently, they calculate macroeconomic impacts of deploying CO_2_ transport and storage (T&S) infrastructure in Scotland using a dynamic CGE, casting doubts on socio-economic benefits.^18^ Patrizio et al. estimate socio-economic impacts of a range of scenarios across SDG indicators including GVA and employment for the power sector, finding both improvements and declines depending on country and decarbonization strategy.^19^ In a subsequent study, they focus on hydrogen as low-carbon fuel as part of the decarbonization strategy and quantify the value creation on a regional level in the UK.^20^

Greater numbers of publications center around the effects of transition scenarios on employment. In particular, there is a body of research focused on the impact of renewable energy on job creation. A study suggests there is a high variation of employment factors for PV, concentrated solar power (CSP), and wind, expected to decrease due to technology learning and economies of scale,^13^ and that hydro and bioenergy have the highest economic impacts among the renewable energy sources,^21^ noting that these are diminished by increased import shares. Several analyses maintain that transformations toward renewable energy offer a gross job creation,^14^^,^^22^ and renewable and low-carbon energy sources generate more jobs per unit energy compared to fossil energy.^15^ A meta-analysis of publications relating to the question of green jobs arrives at “reasonable evidence from the literature that renewable energy and energy efficiency are more labour-intensive than fossil-fired generation”.^23^ The need for an appropriate counterfactual to which to compare a given scenario is emphasized. Whilst analysis of renewable energy specifically is certainly necessary for planning the transition, it should be noted that most energy systems may be comprised of a portfolio of technologies, including intermittent renewables, bioenergy, low-carbon dispatchable power, and negative emissions technologies. Analyses for the entire electricity system are therefore also warranted.

Some studies evaluate the electricity system as a whole and identify sectors with job losses and creation. Using employment factors or job creation by technology, a study calculates employment generated for 50 countries and finds that decarbonization creates jobs compared to a reference, with job losses in fossil resources and job gains in wind and solar.^24^ The Inter-American Development Bank and International Labor Organization also project job losses in fossil electricity and extraction, which are offset by job gains in agriculture, renewable electricity and other sectors, calling for policy to support the reallocation of workers.^25^ A study by the European Commission estimates the impact of the transition on GDP and employment as “small but positive”.^26^ The report emphasizes the need for reskilling, retraining, and reallocating workers. Analysis utilizing an input-output methodology also finds job losses in mining and extraction, refining, and related to coal and natural gas, and job creation in construction, manufacture of electrical parts and machinery, related to renewable power generation, totaling at slightly positive net effects.^27^ They further suggest there may be disparities in location and skill, so incentives may be needed to reallocate jobs. Trajectories will depend on the country, and transitions need to be guided by policy to mitigate negative effects and take advantage of positive effects. A constant world trade structure is assumed, neglecting adjustment effects and productivity increases. Another analysis using a multi-region, multi-sectoral dynamic CGE model quantifies the employment per energy produced for various energy sources. Job reallocation is calculated for a decarbonization scenario, estimating job losses in fossil fuels and energy-intensive industries, opposite job gains in agriculture, construction, and electricity, with narrowly net positive employment.^28^ A crucial factor which is not analyzed sufficiently in literature is the dependence of these scenarios on the overall trajectory of the system. This includes the path to net zero, e.g., abatement, offsets, offshoring, as well as changes in the amount of resources imported vs. domestically produced.

The coal sector has received some attention in this discussion. An EU-centered analysis estimates that the potential of replacing coal jobs with employment in renewable energy (PV, wind, geothermal, bio, carbon capture and sequestration (CCS)) varies by region, and suggests that coal regions can actively contribute to the energy transition with cooperation between the actors—private sector, policy makers, communities—being essential.^29^ A study of the US coal sector suggests employment can be saved by deploying bioenergy with CCS (BECCS), which could even create jobs along its supply chain.^3^ There is a notable scarcity of analysis of the industrial sector, despite the fact that it is a major contributor to both emissions and employment.

When the skill level is included in a calculation of employment based on employment factors of energy activities, a higher level of qualification is forecast for decarbonization scenarios in addition to rising employment levels.^30^ A perhaps understudied aspect is job quality, which—while potentially harder to quantify—should be protected and might be targeted when transforming and creating jobs.

One study alerts to the question of whether a shift of the technology mix toward more labor intensive is desirable in the long-term compared to a more efficient system, recognizing that the main benefit of the transition remains a low/zero carbon economy.^23^ In this context, it has to be pointed out that concurrent with climate change mitigation there are other potentially disruptive economic trends which impact value and job creation in energy and industry, such as automation, and shifting supply chains under global conflict. With regard to the protection of local economies, some argue incentivizing local production and taxing production elsewhere disrupts global supply chains and impedes progress of the transition rather than create jobs.^31^ It is worth noting that while fossil energy vectors—oil, coal, natural gas—are traded internationally, supply chains for components of low-carbon technologies are also global, energy transition pathways therefore remain inherently linked to geopolitics.^32^ Further, access to several scarce resources required for the manufacture of low-carbon technologies, such as lithium, cobalt, iridium, and rare earth metals, is unevenly distributed internationally, potentially leading to the replacement of old with new global dependencies.^31^^,^^32^^,^^33^

Again, there appears to be significant qualitative discussion around the topics of the “just transition”,^34^ the “green state”,^35^ economic growth and job creation associated with the energy transition, and only limited quantitative analysis. Existing work focuses on the power sector, with few sources discussing industry. Most studies analyze employment, without mentioning gross value added. Further, the share of the activities that is sourced domestically vs. imported is not varied sufficiently, and the evolution of the whole system toward local production vs. offshoring is rarely included in the studies. Therefore, with this study, we aim to contribute quantitative analysis to inform the relevant discourse around the transition and value/job creation. We include not only the power sector, but also industry, specifically cement, steel, and refining. We go beyond existing work by connecting our bottom-up model of power and industry with socio-economic data for the UK to estimate the impact of various transition pathways on GVA and employment. Furthermore, we explicitly vary the share of activities that benefit the domestic economy. In the following, we analyze value and job creation per sector, technology, over time, for various scenarios, and compared to a BAU reference case. We further conduct a thought experiment to maximize value added. We hope that this work can add to a factual basis for designing policy with socio-economic benefits.

## Results

This study builds on the extension of the energy systems optimization (ESO) model to the industrial sector.^36^^,^^37^^,^^38^ We utilize the Jobs and Economic Development Impacts (JEDI) methodology^39^ and integrate a calculation of GVA and jobs into our modeling framework. Please see the STAR Methods section at the end of the paper for details on the model, technology options, socio-economic data, pre-processing, and assumptions for the scenarios.

The analysis is centered around four decarbonization scenarios for industry.

• **BAU and offset**: no abatement is deployed in industry; all emissions are offset by BECCS in the power sector.
• **Abate and offset**: abatement is allowed in industry, residual emissions are offset by BECCS. The import and export of cement, steel, and petrochemicals is kept constant.
• **Import and offshore**: industrial emissions are offshored, i.e., at the end of the time horizon all commodities are imported.
• **Abate and export**: low-carbon production of cement, steel, and petrochemicals is increased over time beyond domestic demand, the surplus is exported.

Abatement is always permitted in the power sector, and a net zero target in 2050 for both sectors combined is enforced for all cases.

In addition, we define scenarios with varying local share of production, enabling an empirical assessment of the importance of import dependence. The local share is the degree to which the goods and services required for the technologies—raw materials, machinery, maintenance, *etc.*—benefit the local economy. These scenarios are characterized as follows, please refer to the method details section for details.

• **All local**: almost all goods and services required for power and industry are sourced locally.
• **Partial import**: the local shares are fixed at their current values where available, and at 50% otherwise.
• **High import**: most import shares are fixed at 100%, except for activities such as maintenance.

The all local scenario can be viewed as estimate of the total GVA and jobs induced by a scenario, domestically and abroad, whereas the high import case represents a plausible lower bound on the effects in the local economy.

### Investment vs. maintenance, power vs. industry

**Figure 1:**
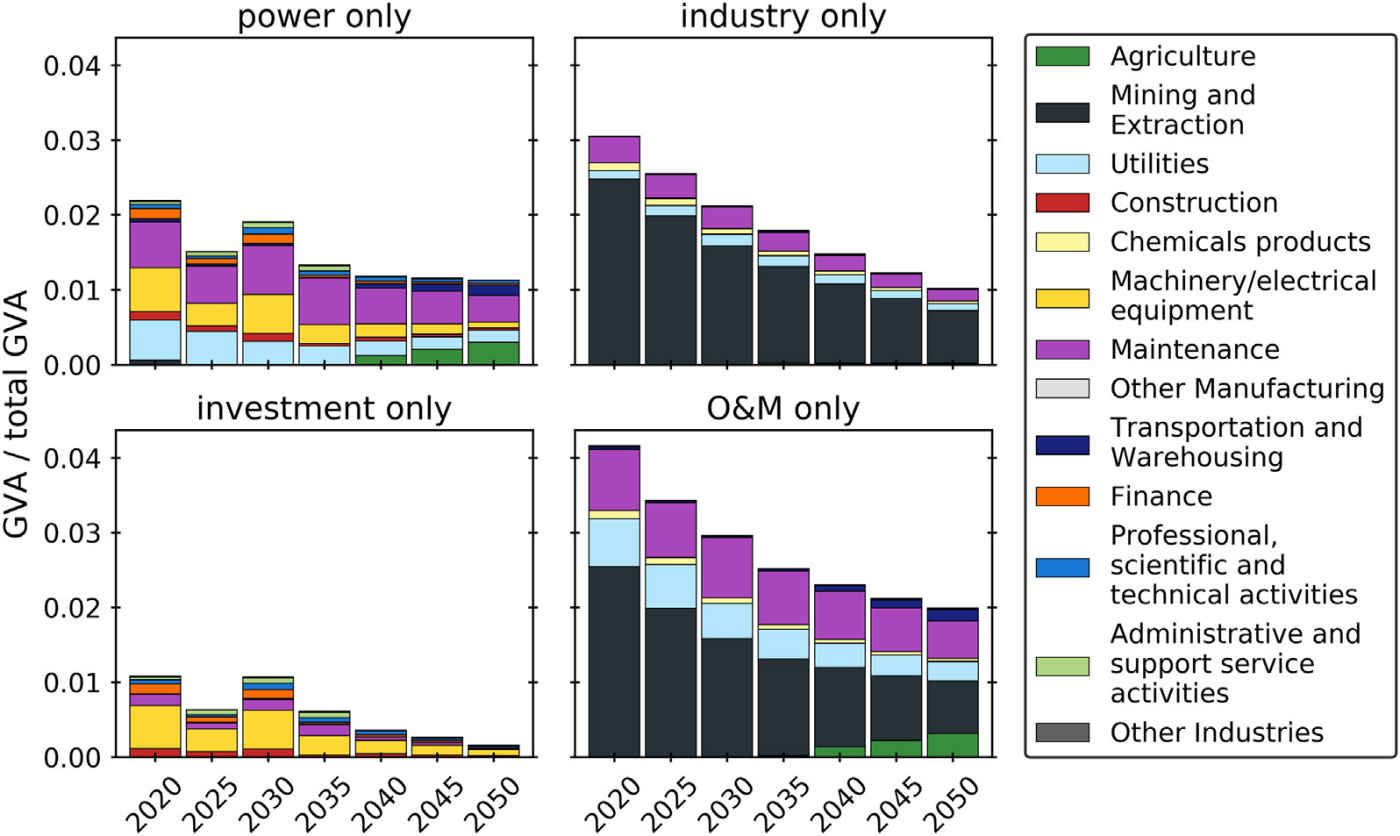
GVA as fraction of total GVA, disaggregated into power and industry (top, investment + O&M combined), and disaggregated into investment and operation (bottom, power + industry combined), by sector and over time for the abate and offset, all local scenario

First, the value creation for the abate and offset scenario combined with the all local assumptions is analyzed. In Figure 1 the total GVA is disaggregated by sector, category, and time. The GVA contribution of power and industry is estimated to be similar, with industry contributing 56% in this scenario. In the power sector, the most GVA is generated in the utilities, machinery, and maintenance sectors. In industry, mining and extraction represents the largest share, with smaller proportions from utilities and maintenance. Operation and maintenance accounts for most of the GVA, around 5 times the total investment GVA. Most of the operation GVA stems from mining and extraction—including coal, crude oil, iron ore, and limestone consumption, as well as utilities—mainly from natural gas consumption, and maintenance. Machinery represents the largest share of investment GVA, followed by construction, maintenance, and finance.

The high GVA in machinery and construction in the early time steps reflects the capacity expansion in the power sector. Utilities GVA decreases over time as a result of the reduced share of gas-fired power. Toward the end of the time horizon GVA in agriculture and transportation increases alongside the deployment of BECCS and industrial CCS.

**Figure 2:**
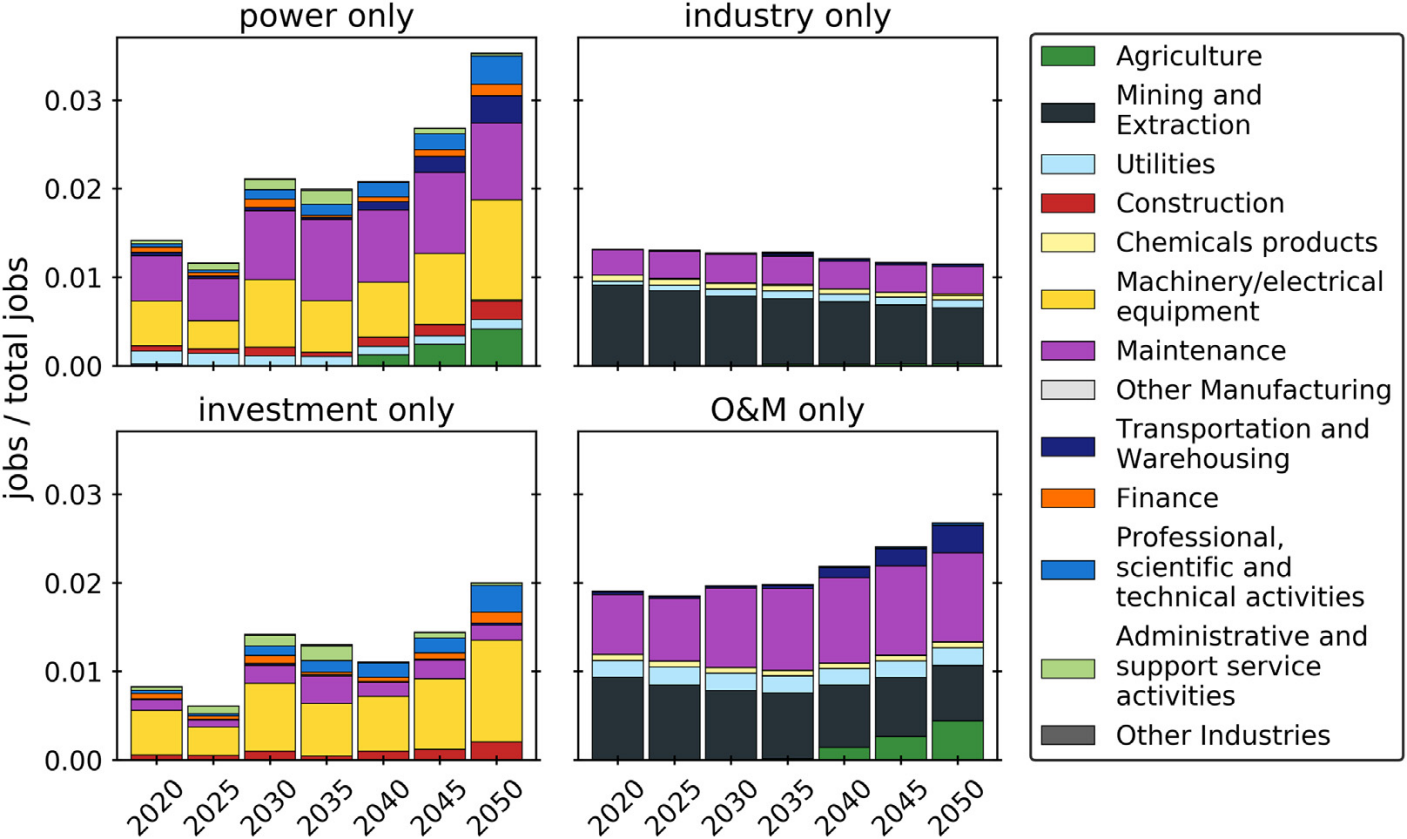
Jobs as fraction of total jobs, disaggregated into power and industry (top, investment + O&M combined), and disaggregated into investment and maintenance (bottom, power + industry combined), by sector and over time for the abate and offset, all local scenario

Figure 2 shows the same breakdowns for job creation. It appears more jobs—around two-thirds of total jobs—are created in the power sector compared to industry, and by O&M—also around two-thirds of total jobs—compared to investment. Annual jobs increase in the power sector and overall, while decreasing slightly in industry. The majority of jobs in industry are maintenance jobs, whereas the jobs in the power sector are a combination of construction and maintenance jobs. The sectors machinery, maintenance, construction, and professional activities contribute the most among the investment jobs. Most of the O&M jobs are within the maintenance and mining sectors, with smaller shares in the utilities and agriculture sectors. The utilities sector notably contributes more to GVA than to jobs. The stable level of investment jobs throughout the decades reflects the constant investment and capacity expansion necessary to achieve the transition. Again, an increase in employment in agriculture and transportation is a result of the deployment of BECCS and industrial CCS.

**Figure 3:**
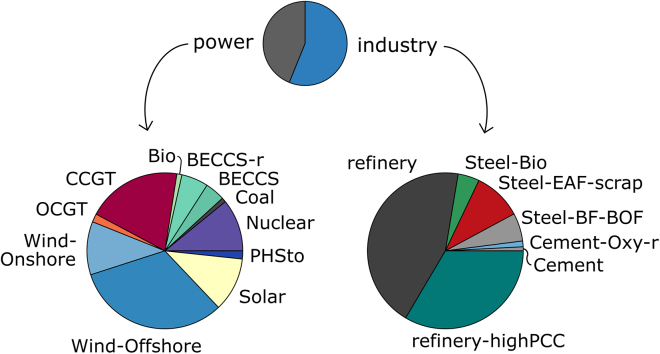
GVA by technology for the abate and offset, all local scenario

**Figure 4:**
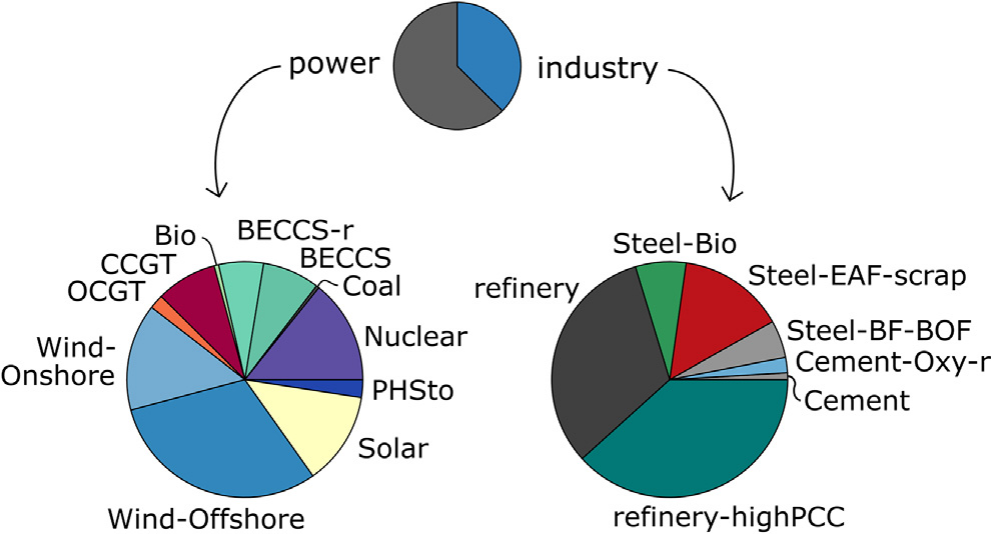
Jobs by technology for the abate and offset, all local scenario

In Figures 3 and 4, the total GVA and total jobs over the time horizon created by individual technologies are detailed. More than half of power GVA and jobs are contributed by intermittent renewable energy sources—solar, onshore and offshore wind, with offshore wind alone accounting for more than 30%. BECCS, new-built and retrofit, is estimated to create similar levels of value and employment as nuclear power, around 10% of GVA and 14% of jobs. Gas-fired power contributes a higher share to GVA compared to jobs relative to the other technologies.

In industry, the refinery sector generates around three-quarters of the GVA and employment. Steel production, conventional, low-carbon, and carbon negative, accounts for most of the remaining quarter, while cement plants create very little value added and jobs. The breakdown within the industrial sectors by technology mirrors the technologies’ shares of production summarized over time.

The total GVA per technology differs by scenario. In the BAU reference scenario (maintaining at most the current level of emissions), CCGT generates 31% of power sector GVA. In the presence of a net zero target, this is displaced by GVA from nuclear and BECCS. In scenarios without abatement in industry (reference/BAU, BAU and offset, import and offshore) the only low-carbon technology in industry is steel-EAF with 10% of GVA. The compositions are very similar among scenarios with abatement in industry (abate and offset, export and offset).

The breakdowns of jobs are similar to the GVA breakdowns. In the reference scenario, almost three-quarters of jobs are generated by intermittent renewable generation capacity. The contribution of Steel-EAF to jobs is higher than to GVA, while CCGT capacity induces less employment than GVA.

When higher import shares are assumed, the relative contributions of offshore wind, nuclear, steel, particularly EAF-scrap, and the cement technologies increase, while those of the other technologies decrease. In most of the scenarios the power sector generates more value (41–79% of total GVA) than industry, and in all scenarios it generates more employment (52%–83% of all jobs) compared to industry.

Analysis of GVA and employment by technology at the end of the time horizon reveals that in the all local case in net zero systems around half of GVA and jobs in 2050 are contributed by BECCS, retrofit and new-built. In scenarios with lower local shares, BECCS represents around a third of total GVA and employment. The next highest contribution in the power sector is offshore wind, with a quarter to a third of GVA and jobs in 2050. When abatement in industry is permitted, almost the entire GVA and job creation in 2050 stems from low-carbon or carbon negative technologies, following the technology mix.

### Value and job creation for varying net zero scenarios

**Figure 5:**
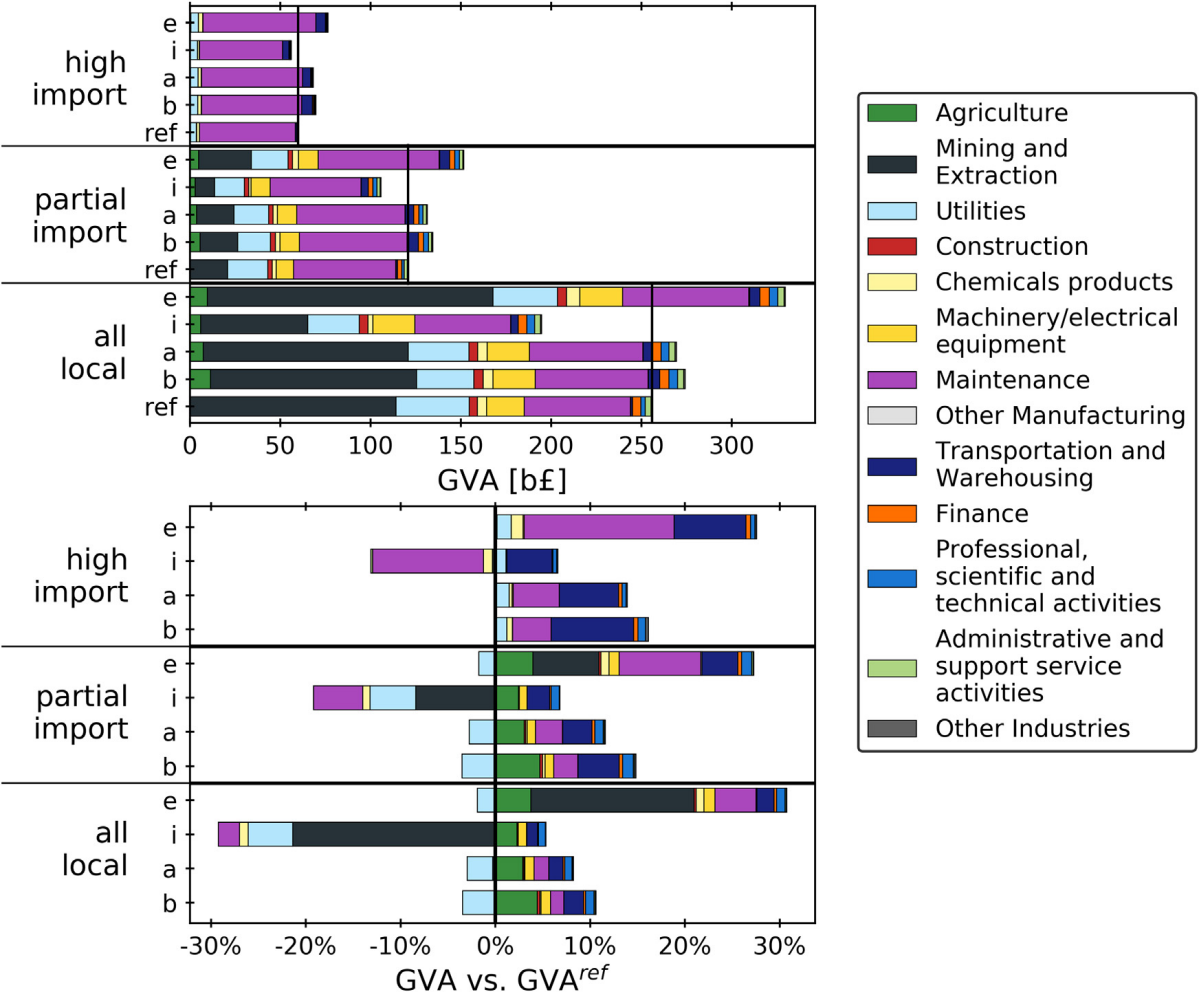
Total GVA and GVA vs. reference/BAU (ref) case for all combinations of import share scenarios (all local, partial import, high import) and industrial sector scenarios (BAU and offset (b), abate and offset (a), import and offshore (i), abate and export (e)). Vertical lines indicate reference scenarios.

In this section, value and job creation are compared across both the trade scenarios for the industrial sector—reference/BAU (ref), BAU and offset (b), abate and offset (a), import and offshore (i), abate and export (e)—and the scenarios for local and import share of the technologies and services—all local, partial import, high import. Figure 5 depicts the total GVA as well as the GVA increase and decrease relative to the respective reference case for all combinations of the scenarios. It is clear that the assumptions around local vs. import share dictate the magnitude of estimated total GVA over the time horizon. In partial import scenarios, total GVA is around half compared to all local, and GVA in the high import scenarios is halved again compared to partial import. The reference scenarios generate roughly 260 b£, 121 b£, and 60 b£ of GVA, for all local, partial, and high import, respectively. Under all local assumptions, the highest contributing sectors are mining and extraction, maintenance, utilities, and machinery/electrical equipment. In scenarios with partial import, GVA in most sectors is reduced, and maintenance provides half the GVA, with mining, utilities, and machinery supplying most of the remaining half. In the case of high import, the bulk of GVA is generated in the maintenance sector, with minor contributions in utilities and transport. This reflects the assumption that fixed OPEX always contributes to the local economy, whereas output toward construction and variable OPEX such as fuel and raw material costs is diminished in the high import scenario.

A comparison of the industrial sector scenarios vs. the respective reference scenario reveals the impact of deploying abatement in power and industry on GVA. The results show a significant increase in GVA when a net zero target is applied and import and export remain unchanged. BAU in industry and offsetting industrial emissions in the power sector (BAU and offset) is estimated to result in a GVA increase of +7%, +11%, and +16%, depending on the scenario. Abating industrial emissions (abate and offset) incurs similar changes in GVA: +5%, +9% and +14%, depending on the scenario. Importing industrial goods and thereby offshoring the emissions (import and offshore) appears to lower GVA by −24%, −12%, and −7%, for all local, partial import, and high import, respectively. In the contrary scenario, abate and export, where an increase in production and export of low-carbon cement, steel, and petrochemicals is presumed, increases in GVA by +29%, +26%, and +28% are estimated.

All scenarios see lower utilization of gas-fired power, reducing GVA in utilities, and the deployment of BECCS, increasing GVA in agriculture in the all local and partial import scenarios. Changes in maintenance GVA are a result of the overall increase or decrease in output. The increase in transportation GVA reflects the deployment of CO_2_ transport and sequestration infrastructure.

**Figure 6:**
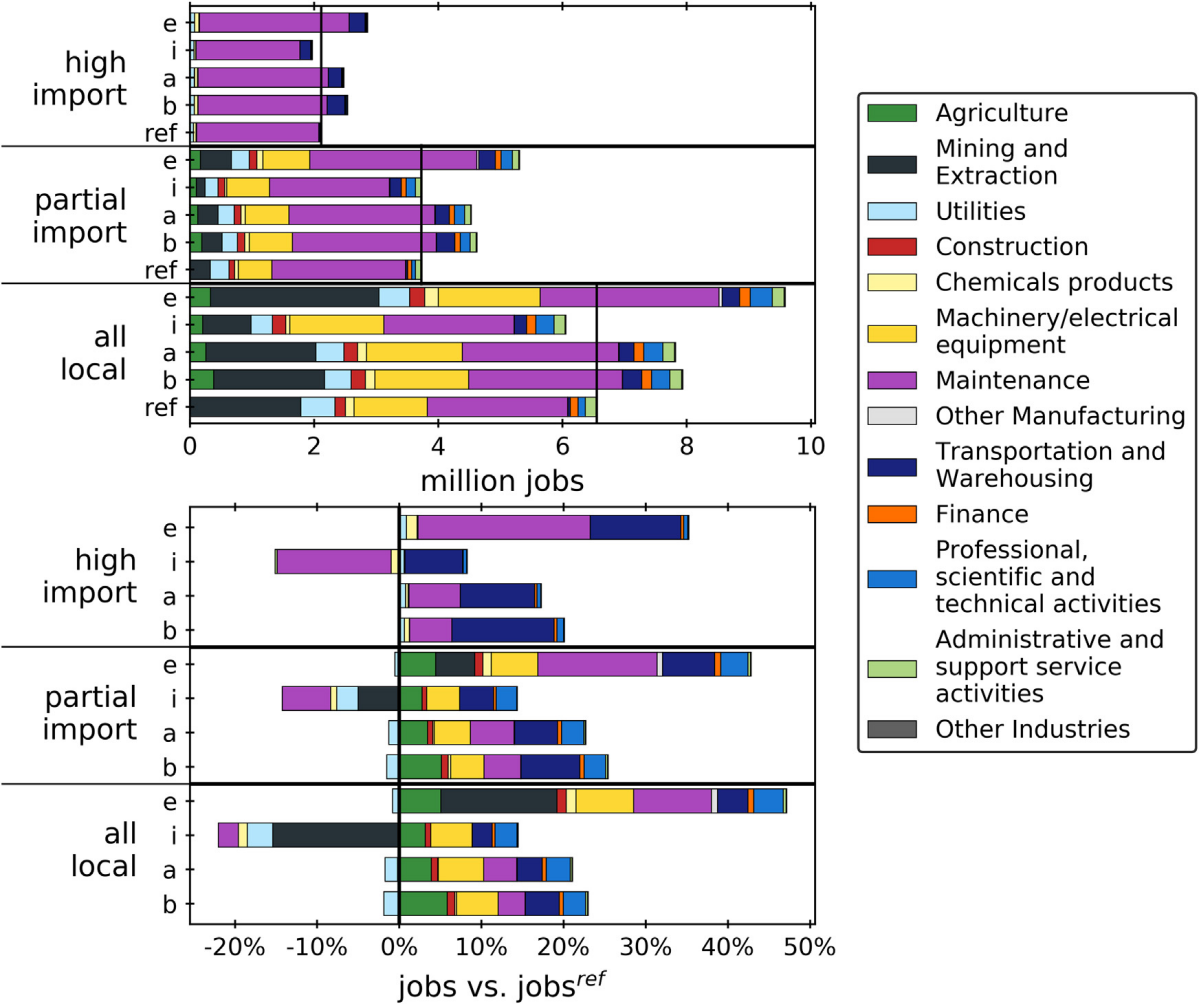
Total employment and employment vs. reference/BAU (ref) case for all combinations of import share scenarios (all local, partial import, high import) and industrial sector scenarios (BAU and offset (b), abate and offset (a), import and offshore (i), abate and export (e)). Vertical lines indicate reference scenarios.

The same comparisons for employment are illustrated in Figure 6. The results mirror the GVA results to an extent. The amount of jobs created is reduced by 43% from the all local to the partial import reference scenario, and by another 43% from partial import to high import. The total employment created throughout the planning time amounts to an estimated 6.6, 3.7, and 2.1 million jobs for the three reference scenarios. When all effects are assumed locally, most jobs are generated in the maintenance, mining, machinery, utilities, and agriculture sectors. The partial import scenario sees substantial reduction in mining and machinery jobs. In the high import case, almost all jobs are attributed to the maintenance sector, with a smaller share in the transportation sector.

When comparing the job creation vs. the reference scenarios, trends similar to GVA can be observed. A net zero target with constant import and export ratios results in an increase in employment amounting to +20–24% in the BAU and offset scenario and +17–21% in the abate and offset scenario. The import and offshore scenario is estimated to reduce overall employment by −7–8% in the all local and high import cases. When assuming a combination of importing industrial goods and partial import, the job creation incurred by the net zero target is outweighed exactly by the loss of jobs that are replaced with imports, resulting in a 0% change compared to the reference scenario. The abate and export scenarios sees an overall increase in employment by +35–46%.

Gains in machinery, maintenance, and professional activities are generally related to building and maintaining capacity in power and industry. The rise in employment in agriculture reflects the operation of a biomass supply chain for BECCS in the power sector and for Steel-Bio. Jobs in the transportation sector are needed to support the CO_2_ transport and sequestration infrastructure.

These results also highlight the fact, that if decarbonization causes supply chains to transform from mostly local to mostly imported, GVA and employment gains can be diminished. If the economy were to import more components and services, even while maintaining domestic industrial production, value added and jobs could be lost. Similarly, if value chains for new low-carbon technologies will be largely imported, their impact on value and job creation will be limited.

**Figure 7:**
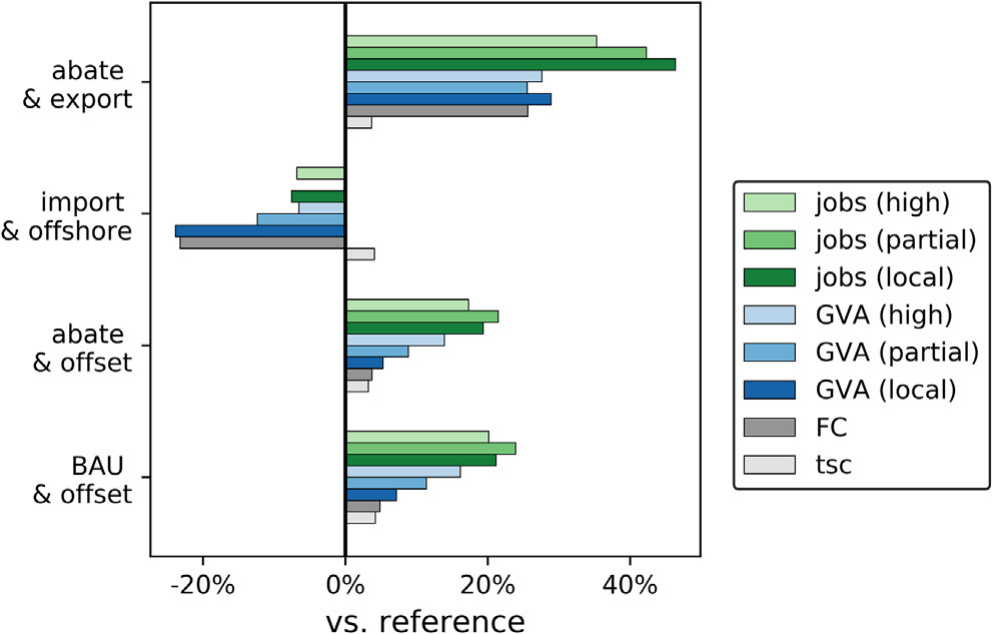
Total system cost (tsc), factor cost (FC), GVA, and jobs relative to the reference (BAU) case for all combinations of industrial sector and import share scenarios

When comparing GVA and jobs for the scenarios, it is helpful to analyze the changes relative to the costs with which they are associated. Figure 7 sums up the total system cost (tsc), factor cost (FC), GVA, and employment for all the scenarios. Factor cost in this context is defined as total system cost without import cost or export revenue from the industrial sectors. Furthermore, value creation and job creation, measured in GVA/tsc and tsc/job, are detailed in Table 1. It is evident that the increase in tsc for all the scenarios is relatively minor at +3–4%, while the factor cost increases slightly for scenarios b and a (+5% and +4%), decreases by −24% for the import scenario, and increases by +26% in the export scenario. In the scenarios which see a rise in GVA and jobs it seems to be higher than the increase in tsc and FC, indicating relatively large socio-economic benefits at relatively low cost. GVA and employment appear to move in parallel with FC to an extent. The import and offshore scenario is estimated to have an increase in tsc similar to the other scenarios, but a reduction in FC as well as GVA and jobs. Increases in job creation seem to be higher than increases in GVA in all cases, although this may be a result of the way GVA and employment are calculated (specifically the use of the end-of-life weighing factor and the discounting).

**Table 1:** Value creation and job creation across scenarios

		GVA/tsc			jobs/tsc (jobs/m£)		
		total	power	industry	total	power	industry
all local	ref.	30.3%	31.5%	29.5%	7.77	10.96	5.65
	b	31.2%	33.4%	29.5%	9.03	13.65	5.65
	a	30.9%	32.8%	29.6%	8.98	13.60	5.73
	i	22.2%	32.8%	14.9%	6.90	13.52	2.38
	e	37.7%	32.9%	41.1%	10.96	13.70	9.01
partial import	ref.	14.3%	21.4%	9.6%	4.42	7.43	2.43
	b	15.3%	23.1%	9.6%	5.26	9.13	2.43
	a	15.1%	22.6%	9.8%	5.20	9.04	2.50
	i	12.0%	22.7%	4.8%	4.25	9.01	1.01
	e	17.3%	22.7%	13.5%	6.07	9.12	3.90
high import	ref.	7.1%	11.2%	4.4%	2.51	3.93	1.56
	b	7.9%	12.7%	4.4%	2.89	4.71	1.56
	a	7.8%	12.6%	4.5%	2.85	4.61	1.60
	i	6.4%	12.5%	2.2%	2.24	4.58	0.65
	e	8.7%	12.5%	6.1%	3.27	4.61	2.31

The GVA/tsc can be regarded as overall indicator for value creation, reflecting the proportion of total cost which generates GVA. The value creation is estimated to be higher in the power sector than the industrial sector in almost every case, ranging from 12.2% to 33.4% compared to 2.2%–41.1%. In the power sector every net zero scenario achieves a higher relative value creation than the reference scenario. The industrial sector exhibits nearly the same GVA/tsc in the reference scenario, BAU and offset, and abate and offset. The import and offshore scenario reduces GVA/tsc by around half, whereas abate and export significantly increases it. Overall, the relative value creation is higher for the b, a, and e scenarios, and lower in scenario i, compared to the reference scenario. Furthermore, reducing the local share decreases GVA/tsc, and appears to have a higher impact in industry compared to power.

Similarly, the power sector is estimated to generate more jobs for the same cost compared to industry. The relative job creation in the power sector is significantly higher for net zero systems compared to BAU. In industry, the relative job creation is similar for BAU and abatement, while importing commodities and exporting low-carbon goods lead to a decrease and increase in jobs/tsc, respectively.

### Maximizing GVA

As the final results in this study, a thought experiment is presented where GVA is increased artificially beyond its value in a system with purely minimized cost. The following equation is added to the model formulation, where GVAdir is the total GVA across all technologies, economic sectors, and time steps, ${\text{GVAdir}}^{\text{ref}}$ denotes the total GVA from a previously concluded cost minimization scenario, and α represents a factor set exogenously:

(Equation 1) $$\text{GVAdir}\geq \left(1+\alpha \right){\text{GVAdir}}^{\text{ref}}$$

The model is then solved with this lower bound on the GVA, minimizing total system cost, for increasing factors α. A net zero carbon target, the abate and offset and the all local assumptions are used in this analysis. The results simulate systems which operate sub-optimally, i.e., are more costly compared to the reference scenario, but achieve a specified increase in GVA at least cost.

In the industrial sector, higher lower bounds on the total GVA lead to later and reduced deployment of abatement. The low-carbon technologies are added to the system later, then first disappear in the cement sector, then the steel sector, and then the refinery sector. At 10% GVA increase, almost the entire capacity in 2050 is still low-carbon, and at 20%, no abatement is deployed at all (beyond EAF-scrap).

**Figure 8:**
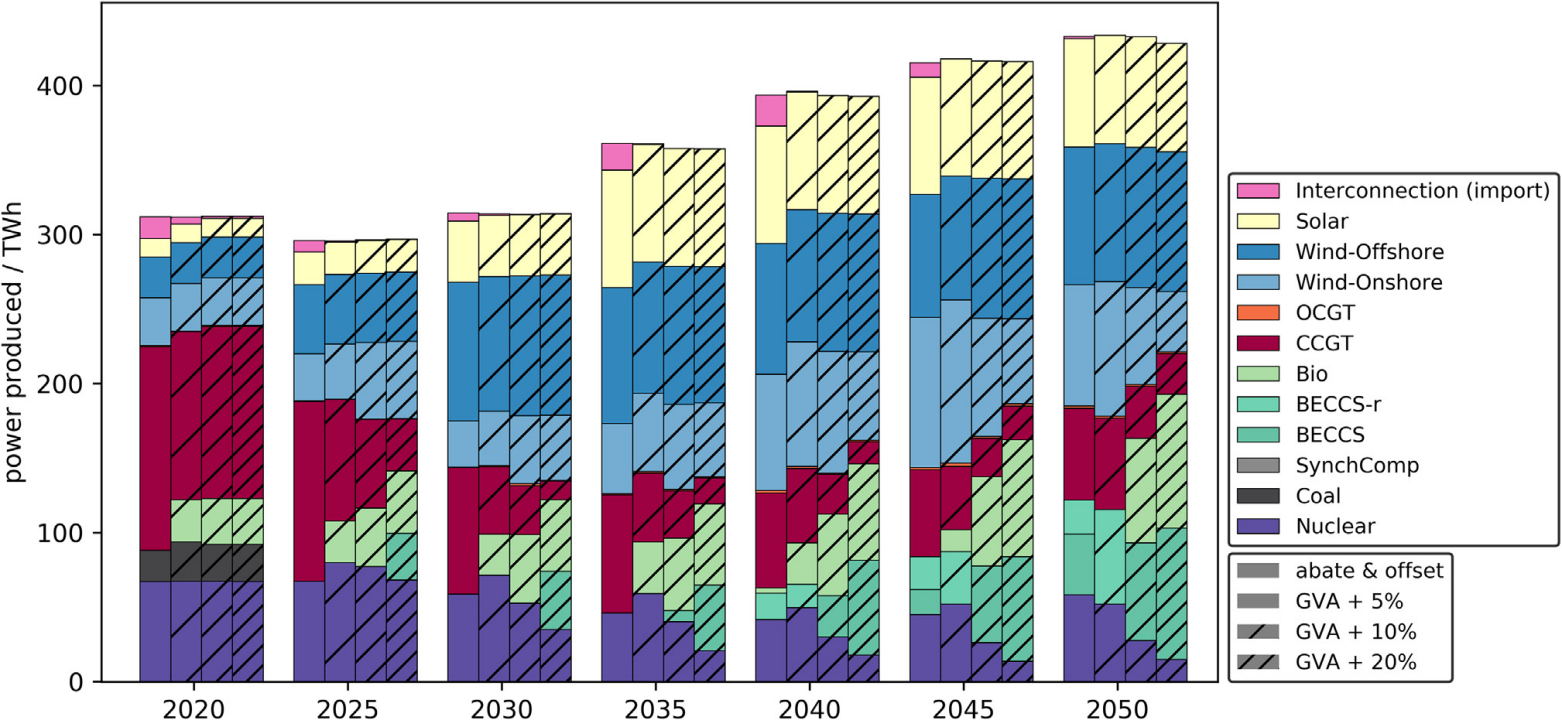
Power produced for abate and offset (all local) scenario with GVA increased by 5%, 10%, and 20%

The results in the power sector are shown in Figure 8 in terms of power produced. In the early decades, substantial amounts of gas-fired power are replaced by bioenergy for all scenarios with increased GVA. At 5% GVA increase, the power production remains the same otherwise. The scenarios with 10% and 20% GVA increase see an earlier deployment of BECCS in addition, as well as higher amounts of electricity generated by bioenergy and BECCS. In these scenarios, bioenergy and BECCS replace substantial fractions of nuclear power, CCGT, and onshore wind generation toward the end of the time horizon. The increased supply of negative emissions allows for the aforementioned reduction of abatement in industry. If a low-carbon industry were enforced, the additional negative emissions could provide offsets for other hard-to-abate sectors.

**Figure 9:**
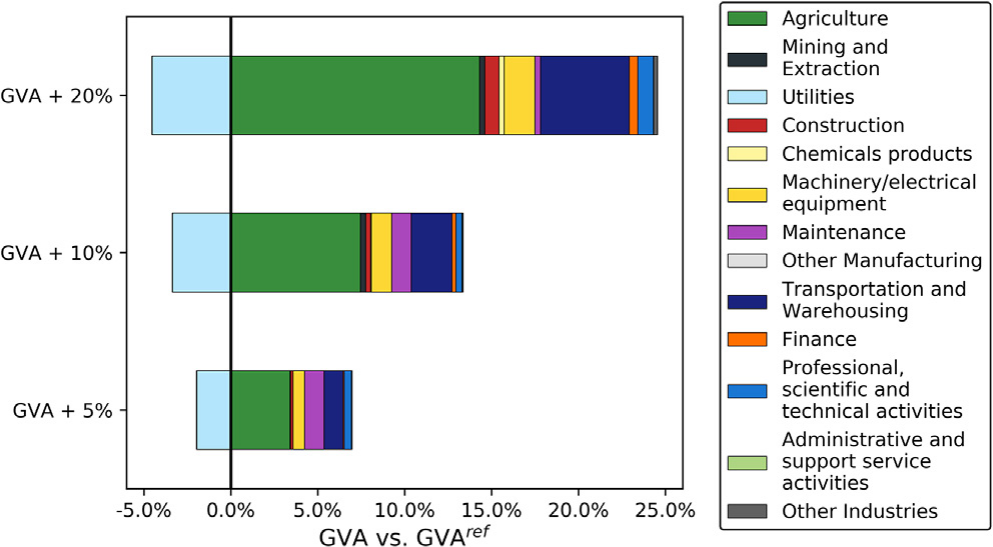
Breakdown of GVA increase relative to abate and offset (all local) for systems with increased GVA

Figure 9 details the breakdown of the GVA increase by sector. GVA in the utilities sector declines with the reduced utilization of gas-fired capacity. The highest GVA gains are achieved in the agriculture sector as a result of the rise in power generation from biomass. The transportation sector also contributes to the higher GVA, reflecting the increased operation of T&S infrastructure for BECCS. At 20% GVA increase, almost half of the power sector GVA and jobs are contributed by bioenergy and BECCS.

The change in system design and operation toward bioenergy and BECCS, away from other power generation technologies including CCGT, under a minimum GVA constraint reflects the relatively high GVA per output in agriculture compared to utilities and other sectors. The biomass supply chain is more complex compared to the natural gas supply chain, resulting in higher value added. The change to BECCS offsets instead of industrial CCS could be a result of the higher GVA/output of BECCS compared to the industrial sector. The minimum GVA requirement shifts the technologies from the most efficient to those which produce higher value per cost. Essentially, the design and operation of a less efficient system creates additional value.

The biomass required for these scenarios, listed in Table 2, increases drastically with increased lower bound on GVA. The biomass used per year in 2050 alone is doubled for the cases with 10% and 20% GVA increase. It reaches 491 TWh/yr in the latter case, which amounts to more than double the estimated indigenous biomass supply of the UK (198 TWh).^40^ The total biomass utilized over the time horizon is multiplied for cases with increased GVA. This could indicate a limit to the extent to which value generation via the biomass supply chain can be expanded in the UK context. It also raises concerns with regard to the viability and overall sustainability of these scenarios. Growing biomass can exacerbate existing environmental burdens including land use, water use, ecotoxicity, and eutrophication. Further, it is expected that the power and industrial sectors will compete with other sectors —heat, chemical industry, negative emissions—for the limited biomass availability.

**Table 2:** GVA, employment, total system cost (tsc) changes, GVA/tsc, total and 2050 biomass consumption for cases with fixed GVA increase

GVA	jobs	tsc	GVA/tsc	total biomass	2050 biomass
269 b£	7.8 m	871 b£	30.9%	1,430 TWh	199 TWh
+5%	+5.5%	+1.1%	32.1%	×2.1	±0%
+10%	+9.4%	+3.5%	32.9%	×3.8	×1.9
+20%	+18%	+9.9%	33.8%	×6.1	×2.5

When comparing GVA, employment, tsc, and GVA/tsc for the scenarios in table Table 2, it becomes apparent that the higher value creation comes at relatively little additional cost. A 10% GVA increase is achieved with only a 3.5% higher tsc. The relative value creation, GVA/tsc, is therefore estimated to rise for higher GVA. Furthermore, higher value creation coincides with higher job creation, in this scenario total jobs increase around as much as GVA.

## Discussion

In this study, socio-economic aspects of the transition to net zero emissions were explored, providing novel quantitative analysis for the power and industry sectors. The JEDI methodology was applied to the model, a corresponding dataset was collected, enabling analysis of GVA and employment for various decarbonization scenarios. Under constant assumptions for trade, the net zero target leads to an increase in GVA and jobs. The estimated gains in value added and employment are slightly higher when industrial emissions are offset by the power sector compared to the case of deploying abatement in industry. Offshoring industrial emissions by reducing the production to zero is shown to reduce GVA and employment, whereas both are significantly increased under the assumption of rising exports from a domestic low-carbon economy. The fraction of expenditure which is assumed to contribute to the local economy is shown to impact the GVA and employment, both can be drastically diminished under high import shares. It is demonstrated that GVA could be increased artificially beyond its value in a system with minimized cost. Under current cost and economic impact assumptions, scenarios with increased GVA and jobs are centered around a sustained deployment of bioenergy and BECCS and a corresponding biomass supply chain.

This work can be expanded in multiple dimensions, some of which arise from the limitations outlined in the next section. GVA and employment multipliers as measure of the structure of the economy have so far been assumed constant over time. It is of interest to estimate time-dependent input-output data, to more accurately model the trajectories of the systems over the decades, and evaluate the impact of large-scale economic trends, such as automation, on the GVA and employment estimates. Incorporating skill levels could shed light on the changing requirements of the transition with regard to the labor force and the potential need for reskilling and reallocating workers. The influence of policy on value and job creation is also of interest, aiming to save and create jobs, or potentially supporting said reskilling and reallocation. Further insights could be gained by comparing the results of this work—utilizing data for the economy as a whole—with bottom-up modeling of job creation.

The estimated value and job creation associated with the transition has implications for industry and policymakers. There are sectors which will experience growth, and sectors which will shrink as the economy moves toward net zero. Companies need to adapt to this reality, and policy is needed to support the transition. It is debatable if the public would support higher prices for products, if they come with domestic employment opportunities. Considering the long lead times for (re)training a workforce, the net zero target has to be taken into account in long-term planning for all industrial sectors. Efforts in the CCS space have been directed at sketching business models for low-carbon technologies. None of these are viable without disincentivising emissions-intensive technologies and supporting new systems. Importing goods with high carbon footprint not just contributes to carbon leakage, but also means less value added and employment for the domestic economy. Again, policy takes on the role of steering the economy toward a desirable direction. It should also be noted that developments in power and industry are not disconnected. The availability of large volumes of renewable power and the development of CCS infrastructure will benefit them both. In the UK, industrial clusters aim to simultaneously promote low-carbon industrial plants and power plants.

Modeling the socio-economic layer of decarbonization reveals connections to larger questions in discussions of climate change mitigation and the economy. Which jobs are worth preserving? What constitutes decent employment? Many industrial clusters in the UK are located in economically challenged areas,^41^ how can it be ensured that the achievements of a low-carbon economic boost benefit these communities^20^? Should paths toward more or less labour-intensive systems be favored?

### Limitations of the study

Several important limitations arise from the design of this study. Most importantly, the structure of the economy is assumed constant. Long-term changes in the economy are therefore not taken into account. If, for instance, overall productivity of the economic sectors were to increase, this may offset some of the job gains. Feedback within the economy and the supply chains is not considered either. Power and industry may compete with other sectors for resources such as biomass as well as skilled workers. Price increases for power and steel have effects on other sectors of the economy. Further, the local share of the technologies is not expected to remain constant over time either (as is assumed in this study). It is conceivable that not only the import share for cement, steel, and petrochemicals will evolve, but so could the share of solar panels, machinery, raw materials, *etc.*, that will be imported. This is especially true if designated policies are implemented. As both GVA and wages are expected to rise with inflation, it should not alter the results. However, if wages relative to output and GVA were to increase, this could impact the estimated job creation. We do not consider induced jobs in this analysis, as this would add significant uncertainty to the model. If these were included, of course the estimates for employment would rise, but so would the associated error bars. Further, the set of technologies included in this modeling somewhat restricts the breadth of the scenarios. Technologies which can provide the same function—low-carbon power, low-carbon steel, negative emissions—at lower cost can displace existing technologies and influence the value creation. Lastly, a carbon price and a carbon target are assumed in this work. The addition of other incentives may change the optimal technology mix and with it the value and job creation. The main insights of this work remain: the net zero transition requires the system to operate slightly less efficiently, as a zero carbon balance is enforced in addition to demand satisfaction. This entails a higher cost and, consequently, higher GVA and employment.

## Resource availability

### Lead contact

Further information and requests for resources should be directed to and will be fulfilled by the lead contact, Caroline Ganzer (cganzer@mpi-magdeburg.mpg.de).

### Materials availability

This study did not generate any new materials.

### Data and code availability

• Socio-economic data are included in the supplemental information. Data will be made available by the lead author upon request.
• The JEDI tool is available from NREL.^39^ The ESO model is available at zenodo.^42^ A complete model formulation is contained in Ganzer, 2022.^38^
• Any additional information required to reanalyze the data reported in this paper is available from the lead contact upon request.

## Acknowledgments

C.G. gratefully acknowledges her PhD studentship funded by 10.13039/501100007185TotalEnergies. N.M.D. acknowledges funding from the UK Engineering and Physical Sciences Research Council (EPSRC) under grant EP/T033940/1.

## Author contributions

C.G.: conceptualization, methodology, data curation, calculations, visualization, writing, editing. P.P.: methodology, data curation. N.M.D.: conceptualization, supervision, editing.

## Declaration of interests

N.M.D. consults widely for public and private sector organizations broadly focused on the net zero transition. P.P. is a member of the advisory board of iScience.

## STAR★Methods

### Key resources table

**Table:** 

REAGENT or RESOURCE	SOURCE	IDENTIFIER
**Deposited data**		

Annual Survey of Hours and Earnings (ASHE), 2016	UK Office for National Statistics (ONS)	https://www.ons.gov.uk/employmentandlabourmarket/peopleinwork/earningsandworkinghours/datasets/ashe1997to2015selectedestimates
Regional Gross Value Added, 2016	UK Office for National Statistics (ONS)	https://www.ons.gov.uk/economy/grossvalueaddedgva
Annual Business Survey, 2016	UK Office for National Statistics (ONS)	https://www.ons.gov.uk/businessindustryandtrade/business/businessservices/datasets/uknonfinancialbusinesseconomyannualbusinesssurveysectionsas

**Software and algorithms**		

Energy Systems Optimization model	Imperial College London	https://doi.org/10.5281/zenodo.1048943
Jobs and Economic Development Impacts model	US National Renewable Energy Laboratory (NREL)	https://www.nrel.gov/analysis/jedi/international.html

### Method details

#### Power and industry

This work is based on the extension of the energy systems optimization framework (ESO) to the industrial sector.^36^^,^^37^^,^^38^ It determines cost-optimal design and operation of the power system and industry. The model simultaneously optimizes capacity expansion from 2020 to 2050, and hourly power generation and storage. It balances the power provided by the generators, power from and to storage, and power demand, which is set exogenously. It determines the power generation and storage capacity decommissioned and built every time period, while adhering to maximum build rates. The model includes constraints on ancillary services – lower bounds on system inertia and reserve capacity. In previous work, a fixed power demand from electrification of heat and transport was added, yielding a higher, more peaky power demand curve. When the industrial sector is connected, its power demand is added to the baseline power demand. The carbon balance is written for both sectors combined, such that industrial emissions and residual emissions in power are offset by negative emissions from BECCS. The complete model formulation is omitted here and can be found in^36^^,^^37^^,^^38^ and the references therein. The model is formulated as mixed-integer linear programming problem (MILP), and solved with CPLEX using GAMS and its Python interface. We have applied the model to the UK context, and considered the cement, steel, and refining sectors; the technologies included in the dataset are listed in the below table. Further, the model features a description of industrial demand and trade. Domestic industrial demand is set exogenously, over time it increases for steel, and decreases for petrochemicals. Then domestic production is balanced with import and export. We estimated the ratio of import to demand, and export to production volume from historic data. This gives rise to a reference, and four archetypal scenarios: the reference scenario assumes no carbon target and no change in import and export. For *BAU&offset*, a net zero carbon target in 2050 with a linear trajectory is applied, but no abatement is available for industry. Here, industrial emissions have to be offset by BECCS in the power sector at the end of the time horizon. The deployment of low-carbon technologies in industry introduces *abate&offset*. In this case, abatement is cost-optimally deployed in every industrial sector, and residual emissions are balanced by BECCS. *Import&offshore* simulates a scenario where industrial production is reduced to zero, and industrial emissions are offshored. Here, domestic capacity is shut down over time, thereby emissions are eliminated, and imports ramp up. Finally, with *abate&export* we imagine a case where abatement is deployed in industry beyond domestic demands, and low-carbon goods are exported. In previous work, we have used this model to explore the role and value of inter-seasonal storage in zero-carbon electricity systems,^36^ examine the interaction between power and industry, and assess the impact of policy instruments on the transition.^37^

**Table:** Technologies included in the model

power	generation	nuclear, coal, bioenergy, BECCS (retrofit & new-built), CCGT, CCGT-CCS (retrofit & new-built), OCGT, Wind-Onshore, Wind-Offshore, Solar
	storage	pumped hydro, battery
	other	interconnection, synchronous compensator
industry	cement	conventional, post-combustion capture (PCC), oxy-combustion, membrane-assisted liquefaction (MAL), calcium looping – tail-end & integrated (CaLtail, CaLint); all retrofit & new-built
	steel	blast furnace - basic oxygen furnace (BF-BOF), electric arc furnace using scrap (EAF), BF-BOF with CCS (retrofit and new-built, low & high capture rate), hydrogen direct reduction (H-DR), H-DR utilizing scrap (H-DR-scrap), steelmaking using biochar with CCS (Steel-Bio), electrowinning (EW)
	refinery	conventional, retrofit post-combustion capture (PCC, low & high capture rate)

#### Estimating GVA and employment for power and industry

For the purposes of this work, the *Jobs and Economic Development Impacts (JEDI)* methodology is applied to the model.^39^ The approach follows previous work with JEDI^3^^,^^19^ to an extent, and is summarized in figure S1 in the supplemental information. JEDI relies on disaggregating a scenario’s economic output by economic sector, and using the value and job creation associated with the sectors to estimate the scenario’s impact. Specifically, the GVA per output for every economic sector is available from socio-economic databases. Using the share of output used for compensation and the average wages, the number of jobs per output for the economic sectors is estimated. Both are included in the model as multipliers $\frac{\text{GVA}}{\text{output}}$ and $\frac{\text{jobs}}{\text{output}}$ – see Table S1 in the supplemental information. We hard-link our model with JEDI, the total system cost (tsc) calculated by the model for power and industry represents the economic output in JEDI. It is comprised of CAPEX of building new capacity, fixed OPEX, and various variable OPEX components, such as fuel cost, in both the power and the industrial sector. In the JEDI context, the costs are separated by category – Inv for investment, i.e., CAPEX, OMF for fixed operating and maintenance costs, and OMV for variable operating and maintenance cost. The output is further disaggregated into its components based on techno-economic analysis in the literature. Inv costs consist of equipment cost, installation cost, building cost, engineering, land, *etc.*, OMF includes maintenance cost and insurance, while fuel and raw material costs are counted toward OMV. All the individual cost/output components are then assigned to the sector of the economy in which they are assumed to generate GVA and jobs. The allocation to 31 OECD sectors is carried out based on the *International Standard Industrial Classification of All Economic Activities (ISIC)* by the UN,^43^ then these sectors are condensed to 15 JEDI sectors (see Table S2 in the supplemental information). The costs of coal, limestone, iron ore, and crude oil, for instance, are assigned to the mining & extraction sector; installation costs and cost of buildings are assigned to the construction sector; *etc.* None of the activities contribute to the sales and ICT sectors, these are therefore dropped in the analysis. Further, for every cost component the *local share* – the fraction of all value that is created in the local economy as opposed to abroad – is estimated and then multiplied by the output. Multiplication of the output and the $\frac{\text{GVA}}{\text{output}}$ and $\frac{\text{jobs}}{\text{output}}$ finally yields the GVA and jobs generated per technology, sector, category, and year. The formulation of the equations is omitted here and available in Ganzer, 2022.^38^

Parts of the total system cost do not generate value or employment and therefore do not enter the GVA/employment calculations. This includes the carbon price paid, negative emissions credit earned, cost of imported power, and the cost and revenue from import and export of industrial products. The cost incurred by the production of the commodities is counted toward GVA and jobs regardless of whether they are sold domestically or exported. Further, only the industrial sectors that have been explicitly modeled (cement, iron and steel, refining) have influence on the GVA and employment.

The socio-economic data for the UK (GVA/output, GVA, compensation, compensation/output, wages) is obtained from the Office for National Statistics (ONS)^44^^,^^45^^,^^46^ for the year 2016, and summarized in Table S1 in the supplemental information. One job in this context refers to one year of full time equivalent (FTE), i.e., full time employment for one person for one year. Within this methodology, only direct jobs are considered, indirect and induced jobs are not estimated. The power sector data are adapted based on a previous study employing ESO and JEDI,^19^ originally obtained from IRENA, NREL, US EIA, and other sources.^47^^,^^48^^,^^49^^,^^50^^,^^51^ Interconnection and synchronous compensator are excluded in this analysis due to a lack of data. Their impact on GVA and jobs is estimated to be minor. Techno-economic data for the industrial sectors appears to be scarce. For the cement sector, the cost disaggregation follows the recent study by SINTEF,^52^^,^^53^ and the economic evaluation therein, which provided the basis for the cost data used in the model.^54^^,^^55^ The breakdowns of capital and operating costs are derived using the published techno-economic data, estimates are added when needed. Electricity is again excluded from the breakdowns since it is accounted for implicitly in the model. In case of steel, the literature sources used for the model^37^ as the basis for the emissions and cost data do not provide cost breakdowns in sufficient detail. Therefore, additional sources with techno-economic analysis are used. For conventional steel production (Steel-BF-BOF), and steel with CCS archetypes (Steel-BF-BOF-CCS-r, Steel-BF-BOF-CCS-n, Steel-BF-BOF-highCCS-r, Steel-BF-BOF-highCCS-n) data are adapted from Hooey et al*.*^56^ A recent report by the IEAGHG on negative emissions technologies^57^ is used as source for cost breakdowns of steel production with biomass (Steel-Bio-r, Steel-Bio-n). Cost disaggregation for steelmaking via direct reduction with hydrogen is performed using data in Jacobasch et al*.*^58^ Gaps in the data are filled with estimates based on similar technologies. Electrowinning (Steel-EW) is excluded from this analysis due to its low technology readiness and corresponding lack of data. There is little data available for the refinery sector. The OPEX breakdown in Robinson^59^ is adapted, and the capital cost breakdown is based on the original source used for cost data.^60^ CO_2_ transport and storage (TS) costs are separated from the operating cost for all technologies and added as a separate technology with a specified cost and cost breakdown. The electricity transmission and distribution is not modeled, its cost and associated value added are not considered in this work. It is expected that value creation from this activity rises throughout the decades, in line with expansion of the power capacity. As the power demand is almost identical among scenarios, it is expected that electricity network infrastructure would add very similar amounts of GVA and jobs in all scenarios.

The scenarios outlined below are usually compared to a reference/BAU scenario. Here, it is assumed that the system maintains at most the current level of emissions, and no other incentives are provided. In this case, retiring capacity in industry is replaced with new-built conventional capacity, except for Steel-EAF, whose share of production increases over time. In the power sector, the capacity mix shifts toward renewable power and gas-fired CCGTs, each providing about half of the total power production in 2050.

The approach detailed above enables an examination of the socio-economic implications of a given decarbonization scenario. It is, however, associated with shortcomings owing to its relatively simplistic nature. One major drawback is the assumption of constant socio-economic data over time. GVA, compensation, wages, are assumed to be constant throughout the time horizon, effectively presuming no changes in the structure of the economy over the decades. In actuality, these indicators are expected to change over time, which would impact the KPI defined here. Projecting these changes and impacts is outside the scope of this study and of interest in future work. Analyzing the implications of automation in manufacturing, for instance, could be particularly interesting. Indirect and induced GVA and jobs are also excluded in this analysis. JEDI grants quantitative insights into GVA/employment outcomes, yet the results are inherently imprecise due to the uncertainties and assumptions in this extension of the model. They are therefore to be treated as estimates revealing tendencies, not as a quantitatively specific guide to the future.

#### Scenarios for local production and import dependence

The fraction of the output attributed to local production vs. production abroad as a function of total production directly impacts the estimated GVA and jobs contributed by a technology in a specific scenario. Certain technologies, such as solar panels and nuclear fuel, are very likely to be imported in a UK context. The maintenance included in the fixed OPEX, on the other hand, is most certainly always entirely local. In order to analyze the effects of varying local share of production and import dependence, several scenarios are constructed. In the *all local* case, almost all effects are assumed to generate GVA and jobs locally. It can be considered an optimistic scenario from the UK’s perspective. It is also a way to estimate the total impact, local and abroad, of a transition scenario. For the *partial import* scenario, parts of the technologies and services are assumed to be imported, reducing the locally induced effects. The *high import* scenario presents a pessimistic case in which most local shares are zero. The below table contains an overview of the three scenarios and the local shares assumed in the power and industry sectors and the subsectors and technologies.

The UK currently depends on imports for 48% of natural gas, 89% of coal, and 83% of crude oil consumption.^19^^,^^61^^,^^62^ These fractions are used for the partial import scenario. The local shares are set to 0 in the high import and 100% in the all local scenario. Significant shares of capacity of solar, onshore wind, nuclear power, and batteries are also currently imported, representing the partial import scenario. For many technologies there is a lack of data concerning the local share and import dependence. For those, a local share of 50% is estimated for the partial import scenario, whereas the high import scenario assumes complete import. Expenditure toward CO_2_ transport and sequestration infrastructure is assumed to entirely benefit the local economy. Again, these numbers are to be understood as estimates, enabling an analysis of systems with mostly local sourcing vs. systems heavily dependent on imports.

**Table:** Assumptions for scenarios for local share and import dependence

			local share			Reference
			all local	partial import	high import	
**industrial sector**						
cement	construction		100%	50%	0%	
	OMF		100%	100%	100%	
	OMV	coal	100%	11%	0%	Patrizio et al.^19^; International Energy Agency^61^
		other	100%	100%	100%	
steel	construction		100%	50%	0%	
	OMV	fluxes	100%	50%	0%	
		ore	100%	50%	0%	
		scrap	100%	50%	0%	
		energy: coal	100%	11%	0%	Patrizio et al.^19^; International Energy Agency^61^
		energy: NG	100%	52%	0%	Patrizio et al.^19^; International Energy Agency^61^
		biomass	100%	50%	0%	
		other	100%	100%	100%	
refineries	construction		100%	50%	0%	
	OMV	crude	100%	17%	0%	UK Department for Business, Energy & Industrial Strategy (BEIS)^62^
		fuel oil & gas	100%	17%	0%	UK Department for Business, Energy & Industrial Strategy (BEIS)^62^
		other	100%	100%	100%	

**power sector**						
solar	construction	hardware	100%	varies	0%	Patrizio et al.^19^; Fu et al.^49^^,^^63^^,^^64^
		installation	100%	50%	0%	
	OM		100%	100%	100%	
onshore wind	construction	turbine module	100%	varies	0%	Patrizio et al.^19^^,^^64^
		other	100%	50%	0%	
	OM		100%	100%	100%	
nuclear	construction	island & project	100%	varies	0%	Patrizio et al.^19^; World Nuclear Association^51^
		other	100%	50%	0%	
	OM	fuel	0%	0%	0%	^65^
		other	100%	100%	100%	
battery	construction	battery	100%	52%	0%	Patrizio et al.^19^^,^^66^
		other	100%	50%	0%	
	OM		100%	100%	100%	
other	construction		100%	50%	0%	
	OM	coal	100%	11%	0%	Patrizio et al.^19^; International Energy Agency^61^
		NG	100%	52%	0%	Patrizio et al.^19^; International Energy Agency^61^
		other	100%	100%	100%	

### Quantification and statistical analysis

There are no quantification or statistical analyses to include in this study.
