# Supplementary material for: Net zero in power and industry—creating or destroying value and jobs?
Source: iScience. 2025 Mar 10;28(4):112185. doi: 10.1016/j.isci.2025.112185 (PMC12018210; doi:10.1016/j.isci.2025.112185)
Supplement: Document S1. Figure S1 and Tables S1 and S2 [file mmc1.pdf]

**iScience, Volume *28***

## Supplemental information

# Net zero in power and industry—creating or destroying value and jobs?

**Caroline Ganzer, Piera Patrizio, and Niall Mac Dowell**

## SUPPLEMENTAL INFORMATION

### Methodology

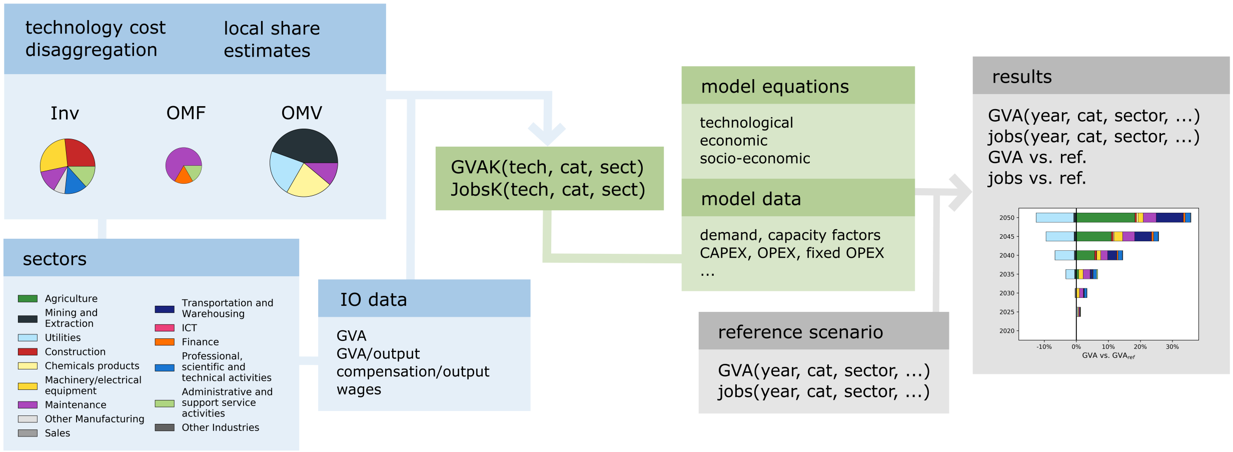


**Figure S1.** JEDI methodology. In pre-processing, expenditure is disaggregated, assigned to economic sectors, and multiplied by the estimated share of local production. Based on input/output data from socio-economic databases, GVA and job creation per economic sector is calculated. Combining disaggregated output and impacts per output yields the multipliers $(\text{GVAK}(\frac{\text{GVA}}{\text{output}}), \text{JobsK}(\frac{\text{jobs}}{\text{output}}))$ for each technology. These are then hard-linked with the calculations of the power+industry model. Results, per sector, time, and overall, are reported in total and in comparison to reference case.

### Socio-economic data

Table S1 contains the gross value added (GVA), GVA as a fraction of output, compensation, wages, compensation as a fraction of output, and the resulting jobs per output for the UK for 2016. As the socio-economic data is published for 31 OECD sectors, they are condensed to 15 JEDI sectors using table S2.

**Table S1.** Input/output data for the UK [S1][S2][S3].

| OECD code | OECD sector | GVA/output m£/m£ | GVA m£ | compensation m£ | wages £/yr | comp./GVA m£/m£ | jobs/output jobs/m£ |
|---|---|---|---|---|---|---|---|
| 1 | Agriculture, forestry and fishing | 46% | 12,064 | 4,419 | 22,145 | 37% | 7.69 |
| 2 | Mining and quarrying | 39% | 11,511 | 4,773 | 38,795 | 41% | 4.16 |
| 3 | Manufacture of food, beverages and tobacco | 28% | 28,881 | 17,009 | 28,683 | 59% | 5.82 |
| 4 | Manufacture of textiles, wearing apparel and leather | 36% | 5,558 | 3,659 | 28,683 | 66% | 8.35 |
| 5 | Manufacture of wood and paper products and printing | 37% | 13,095 | 8,600 | 28,683 | 66% | 8.54 |
| 6 | Manufacture of coke, refined petroleum and chemicals | 18% | 13,379 | 7,004 | 28,683 | 52% | 3.24 |
| 7 | Manufacture of pharmaceutical products | 44% | 13,360 | 4,866 | 28,683 | 36% | 5.59 |
| 8 | Manufacture of rubber, plastic and non-metallic minerals | 37% | 13,669 | 9,436 | 28,683 | 69% | 9.01 |
| 9 | Manufacture of basic and fabricated metal products | 35% | 19,957 | 14,444 | 28,683 | 72% | 8.92 |
| 10 | Manufacture of computer, electronic and optical products | 44% | 12,527 | 7,293 | 28,683 | 58% | 8.91 |
| 11 | Manufacture of electrical equipment | 35% | 4,697 | 3,735 | 28,683 | 80% | 9.68 |
| 12 | Manufacture of machinery and equipment | 34% | 14,085 | 9,273 | 28,683 | 66% | 7.78 |
| 13 | Manufacture of transport equipment | 22% | 26,319 | 15,270 | 28,683 | 58% | 4.35 |
| 14 | Other manufacturing, repair and installation | 40% | 16,021 | 11,072 | 28,683 | 69% | 9.67 |
| 15 | Electricity, gas, steam and air-conditioning supply | 24% | 26,372 | 7,260 | 37,311 | 28% | 1.74 |
| 16 | Water supply; sewerage and waste management | 49% | 23,117 | 7,750 | 29,480 | 34% | 5.62 |
| 17 | Construction | 35% | 108,281 | 48,524 | 30,350 | 45% | 5.23 |
| 18 | Wholesale and retail trade; repair of motor vehicles | 14% | 185,958 | 120,248 | 23,075 | 65% | 4.04 |
| 19 | Transportation and storage | 40% | 74,244 | 53,190 | 28,784 | 72% | 10.02 |
| 20 | Accommodation and food service activities | 54% | 50,023 | 34,832 | 18,206 | 70% | 20.70 |
| 21 | Information and communication | 50% | 117,227 | 68,693 | 38,001 | 59% | 7.77 |
| 22 | Financial and insurance activities | 40% | 128,405 | 68,454 | 38,886 | 53% | 5.48 |
| 23 | Real estate activities | 68% | 249,199 | 14,550 | 27,170 | 6% | 1.45 |
| 24 | Professional, scientific and technical activities | 54% | 134,860 | 83,262 | 34,426 | 62% | 9.75 |
| 25 | Administrative and support service activities | 57% | 90,028 | 56,299 | 24,109 | 63% | 14.82 |
| 26 | Public administration and defence | 40% | 86,795 | 61,623 | 31,914 | 71% | 8.90 |
| 27 | Education | 53% | 102,378 | 83,479 | 30,347 | 82% | 14.26 |
| 28 | Human health and social work activities | 64% | 133,621 | 103,740 | 25,363 | 78% | 19.69 |
| 29 | Arts, entertainment and recreation | 29% | 28,079 | 15,408 | 23,171 | 55% | 6.89 |
| 30 | Other service activities | 48% | 29,463 | 14,598 | 24,870 | 50% | 9.57 |
| 31 | Activities of households | 54% | 4,961 | 4,961 | 1 | 100% | |

**Table S2.** Assignment of OECD sectors to JEDI sectors.

| JEDI code | JEDI sector | OECD code | OECD sector |
|---|---|---|---|
| 1 | Agriculture | 1 | Agriculture, forestry and fishing |
| 2 | Mining and Extraction | 2 | Mining and quarrying |
| 3 | Utilities | 15 | Electricity, gas, steam and air-conditioning supply |
| | | 16 | Water supply; sewerage and waste management |
| 4 | Construction | 17 | Construction |
| 5 | Chemicals products | 6 | Manufacture of coke, refined petroleum and chemicals |
| 6 | Machinery/electrical equipment | 11 | Manufacture of electrical equipment |
| | | 12 | Manufacture of machinery and equipment |
| 7 | Maintenance | 14 | Other manufacturing, repair and installation |
| 8 | Other Manufacturing | 3 | Manufacture of food, beverages and tobacco |
| | | 4 | Manufacture of textiles, wearing apparel and leather |
| | | 5 | Manufacture of wood and paper products and printing |
| | | 7 | Manufacture of pharmaceutical products |
| | | 8 | Manufacture of rubber, plastic and non-metallic minerals |
| | | 9 | Manufacture of basic and fabricated metal products |
| | | 10 | Manufacture of computer, electronic and optical products |
| | | 13 | Manufacture of transport equipment |
| 9 | Sales | 18 | Wholesale and retail trade; repair of motor vehicles |
| 10 | Transportation and Warehousing | 19 | Transportation and storage |
| 11 | ICT | 21 | Information and communication |
| 12 | Finance | 22 | Financial and insurance activities |
| 13 | Professional, scientific and technical activities | 24 | Professional, scientific and technical activities |
| 14 | Administrative and support service activities | 25 | Administrative and support service activities |
| 15 | Other Industries | 20 | Accommodation and food service activities |
| | | 23 | Real estate activities |
| | | 26 | Public administration and defence |
| | | 27 | Education |
| | | 28 | Human health and social work activities |
| | | 29 | Arts, entertainment and recreation |
| | | 30 | Other service activities |
| | | 31 | Activities of households |

## REFERENCES


[1] UK Office for National Statistics (ONS). Annual Survey of Hours and Earnings (ASHE), 2016.

[2] UK Office for National Statistics (ONS). Regional Gross Value Added, 2016.

[3] UK Office for National Statistics (ONS). Annual Business Survey (ABS), 2017.
